# Sport facilities in urban contexts: a multi-perspective discussion

**DOI:** 10.3389/fspor.2026.1882349

**Published:** 2026-10-19

**Authors:** Karin Book

**Affiliations:** Department of Sport Sciences, Malmö University, Malmö, Sweden

**Keywords:** sport facilities, sport infrastructure, governance, urban planning, path dependency, institutional theory, organised sport, Sweden

## Abstract

**Introduction:**

Sport facility provision has become an increasingly prominent issue in urban governance, frequently framed as a growing shortage with implications for participation and public health. However, research suggests that the relationship between sport facility provision, accessibility, and participation is complex and context-dependent. This article explores how sport facility provision in urban Sweden is shaped by different perspectives, competing institutional logics, and fragmented governance arrangements.

**Methods:**

The study adopts an exploratory qualitative design combining document analysis with semi-structured interviews with key informants from national sport organisations, municipal-sector organisations, and knowledge-producing bodies. An abductive analytical approach is applied, iterating between empirical insights and theoretical perspectives, particularly path dependency and new institutional theory.

**Results and discussion:**

The findings reveal a contested policy field in which the notion of “facility shortage” is widely acknowledged but interpreted in divergent ways. Rather than indicating a simple lack of infrastructure, shortage emerges as a multidimensional phenomenon shaped by structural mismatches between needs, demand and provision, institutional inertia, and evolving sport practices. Historical investments and standardised facility models contribute to path-dependent development trajectories, while fragmented governance arrangements complicate coordination and prioritisation. At the same time, increasing reliance on quantitative indicators risks obscuring qualitative dimensions such as usability, inclusion, and social value. The article concludes that sport facility challenges cannot be reduced to increasing supply. Instead, addressing them requires a more reflexive and context-sensitive approach to governance, acknowledging institutional legacies, competing priorities, and changing patterns of sport participation.

## 1 Introduction

One in four sport clubs has a major need for more time or space for their activities, and just as many are forced to turn down new members because of this […]. Historically, access to sport facilities and environments has been good, enabling a broad and successful sport movement. However, conditions have changed over time […]. My message to policymakers in this election year is clear—implement a long-term national investment in sport facilities and environments. I propose 600 million annually. Together, we can get more children active. Together, we can build a stronger Sweden.

This statement was written by the Chair of the Swedish Sports Confederation (RF), Anna Iwarsson, in an op-ed published in *Aftonbladet* on 6 May ((1), translated from Swedish).

“There is a shortage of sport facilities in the country's major cities—so severe that the government is now beginning to take an increasingly active role in addressing the problem.”

This statement opened an article based on an interview with the Minister for Social Affairs with responsibility for sport on the news site *Idrottsbranschen* on 8 April 2026 ((2), translated from Swedish).

Together, these statements reflect a strong and frequently articulated narrative in contemporary Sweden: that sport facilities are insufficient and that existing infrastructure is increasingly inadequate [e.g., (3, 4)]. However, the situation is far from clear-cut.

While substantial national government funding is allocated annually to the sport movement through the Swedish Sports Confederation, these funds have historically not been earmarked for the construction of sport facilities (5, 6). Instead, responsibility for planning, financing, and constructing sport facilities has largely rested with municipalities (7). This institutional arrangement raises important questions concerning priority-setting, resource allocation, and the distribution of influence among actors involved in sport facility provision.

In the post-war period, strong public support—primarily at the local level—underpinned the development of sport facilities, reflecting the central role of sport in Nordic welfare policy. The close relationship between public authorities and voluntary sport organisations has been instrumental in this development and distinguishes the Nordic model from systems in countries such as the United Kingdom and the United States (8). Despite relatively high levels of provision, structural challenges have long been identified. As Rafoss and Troelsen (8) argue, sport facilities are often unevenly distributed, outdated, mono-functional, or designed primarily for competitive sport, making them less suited to contemporary patterns of participation.

Recent urban development trends—particularly urbanisation and densification—further complicate these dynamics. Densification increases competition for urban land and influences how, and to what extent, sport facilities are prioritised in planning processes (9). At the same time, it stimulates the development of new approaches to sport and physical activity, including more compact, multifunctional, and shared-use solutions. Thus, the growing demand for sport facilities expressed by the sport movement coincides with increasing spatial constraints and diverging interpretations of need.

Sport participation depends on access to spaces and facilities. However, these spaces are embedded in complex governance arrangements and subject to competing interests. Different actors occupy different positions within the sport facility policy field and therefore may frame facility needs in different ways. On the one hand, actors within the sport movement stress a need for increased provision; on the other hand, urban planners and municipalities must balance this demand against other societal priorities. As a result, sport facility provision emerges as a multidimensional governance issue shaped by institutional structures, competing logics, and shifting policy priorities. Consequently, the issue of sport facilities in urban environments must be analysed, problematised, and nuanced in order to develop relevant strategies for sustainable, functional, and accessible provision (10).

Against this backdrop, the aim of this article is to explore how sport facility provision in urban Sweden is shaped by different perspectives, competing institutional logics, and fragmented governance arrangements, as articulated by key actors across the sport movement, municipal-sector organisations, and knowledge-producing bodies.

## 2 Research background

The study is situated at the intersection of several research fields, including sport infrastructure provision, urban development trends such as densification, and governance perspectives related to these processes. It is not possible to provide a comprehensive review of all these areas; instead, a selective overview is offered.

A central point of departure for this study is the perceived shortage of sport facilities and the differing perspectives on this issue. As noted above, there is a strong narrative suggesting, on the one hand, that a significant shortage exists and, on the other, that this shortage leads to a range of negative consequences. This narrative is advanced by representatives of sport clubs in television reports and opinion pieces, on the websites of sport organisations, and by the national government. The negative consequences most frequently highlighted include that clubs are forced to turn away prospective members, contributing to increased sedentary behaviour and, in the longer term, to deteriorating public health in the form of both physical and mental ill-health. It is also argued that these developments may lead to insecurity, social exclusion, and potentially increased criminality [see, e.g., (11)].

Thus, access to sport facilities constitutes a central point of departure for this article, even though the aim is not to measure or objectively assess the actual level of provision or its consequences.

Not only in Sweden but also elsewhere, facility provision is a central issue. An international comparative study by Seippel et al. (12) examined problem perceptions among sport clubs in nine countries, identifying facility availability as a common concern.

### 2.1 Availability, accessibility and proximity

Over the past two decades, sport facilities in urban contexts have been examined from a range of perspectives. Common approaches include spatial distribution analyses, which examine facility location in relation to population density and accessibility [e.g., (13–15)], as well as studies of both objective and subjective accessibility [e.g., (16, 17)]. It is therefore not surprising that facilities are frequently discussed in terms of availability, accessibility and proximity, as these aspects are often considered crucial. According to Karusisi et al. (18), spatial accessibility to sport facilities is a key concern for multiple stakeholders involved in policymaking across transport, urban planning, marketing, and public health. The relationship between sport infrastructure and participation is well researched [e.g., (19)], although findings are mixed. Hence, while facilities and physical environments are important—arguably even decisive—for enabling physical activity, this relationship is complex.

Faskunger and Sjöblom (20), referring to Parks, Houseman, and Brownson (21), argue that access to facilities and physical activity appears to follow a dose–response relationship; that is, the more facilities and spaces available in a neighbourhood, the higher the level of physical activity among residents, for adults as well as adolescents and children. Hallmann et al. (22), as well as Wicker et al. (23), conclude that the availability, accessibility and quality of sport facilities significantly influence participation rates in organised sport.

Hoekman, Breedveld and Kraaykamp (15) offer a somewhat different perspective:

Sport policies generally assume that differences in the accessibility of sport facilities are at least partly responsible for differences in sport participation […]. Empirical evidence for this assumption is rather meagre, however, especially in Western and Northern Europe. (p. 205)

Drawing on the Dutch context, the authors demonstrate that expansion of the sport facility stock explained rising participation up to a certain threshold, which was reached in the 1980s. Since then, participation has plateaued and, in some cases, declined. This pattern is observable in several countries and suggests that increasing the number of facilities does not automatically lead to higher participation. Instead, broader considerations relating to changing needs, trends and forms of accessibility must be taken into account.

Another Dutch study, by Prins et al. (24), found that leisure-time sport participation is associated with neighbourhood social capital rather than the availability of parks or sport facilities. More generally, research has tended to focus on physical environmental factors, but findings remain inconsistent: some studies identify positive associations between facility availability and physical activity, whereas others do not. This suggests that the physical environment alone may not sufficiently motivate participation. Rather, social environmental factors—such as social capital—may play an important complementary role.

By contrast, an Australian study (25), based on comprehensive data from a large sub-national region, demonstrates an association between participation in four popular sport and the provision of corresponding facilities, although some of these relationships weaken when controlling for socioeconomic status. As noted above, findings vary across both facility types and forms of sport participation. A German study similarly identified activity-specific differences, showing that swimming pools are important for overall sport participation, whereas sport fields are primarily linked to club-based participation (26).

As emphasised by Karusisi et al. (18), many studies rely on aggregate measures of both accessibility and activity, combining different types of facilities and sports. Evidence from the RECORD Study shows that only accessibility to swimming pools is significantly associated with corresponding activity, while no such effects are observed for team sports, racket sports, or fitness. This indicates that accessibility may matter more for specific activities than for overall participation. In addition, socioeconomic factors such as education and income influence certain forms of activity, highlighting the importance of contextual and individual factors (18).

Eime et al. (25) further note that studies employ both subjective and objective measures when analysing the relationship between physical activity and the presence of sport facilities, with mixed results. It is therefore evident that investing in sport and recreation infrastructure—whether traditional sport facilities or nature-based opportunities—is only one aspect; ensuring accessibility is another. Several studies have linked physical activity to factors such as proximity and travel time to facilities or public spaces, although findings remain inconsistent [e.g., (17, 27, 28)]. Moreover, features such as safety, amenities, maintenance, aesthetics, environmental comfort, walking paths, human scale and landscape qualities are all important in supporting physical activity [e.g., (29–31)]. Zhao et al. (32), drawing on Stimulus–Organism–Response (SOR) theory, identify availability, accessibility, design, safety, and affordability as key factors influencing attitudes towards sport participation and, in turn, behavioural intentions, while also emphasising the role of additional factors. In a study of public, informal outdoor sport spaces, Book and Högdahl (33) highlight aspects such as spatial design, diversity of activities, social support, and perceptions of inclusion and belonging as central to perceived accessibility.

Not only availability but also proximity to facilities is often assumed to be crucial for engagement in physical activity. The Swedish Sports Confederation (RF), for example, claimed in 2023 at its website (34) that individuals with good access to sport facilities are 43 per cent more likely to exercise regularly than those with poorer access. While this figure appears to offer strong support for increased provision, it is taken out of context. It originates from Faskunger and Sjöblom (20), who briefly refer to research by Giles-Corti and Donovan (35) conducted in Australia, focusing on adult populations. Moreover, Giles-Corti and Donovan emphasise that the influence of the physical environment on physical activity is relatively limited compared with individual and social determinants:

Thus, perhaps the most striking finding of this study was that the influence of the physical environment on physical activity levels generally was relatively weak and secondary to individual and social environmental determinants. (p. 1809)

Eime et al. (25) highlight that there is growing evidence that there are differences in sport participation according to residential location, but this is not necessarily related to proximity to sport facilities.

### 2.2 Traditions, standardisation and utilisation

Traditional sport facilities often clash with contemporary urban planning ideals, which emphasise efficiency, sustainability, and economic viability. The study Sport in Spatial Planning, commissioned by the Swedish Sports Confederation, explored the role of organised sport in urban planning and revealed growing difficulties in securing influence and prioritisation within planning processes (36). It highlighted the need for sport clubs to adopt new spatial strategies, though the sport movement, rooted in strong traditions, tends to operate in a monolithic manner. This relates to another important perspective influencing both the provision and perceived availability of facilities, as well as their use and usability, namely the standardisation of sport facilities.

Rafoss and Troelsen (8), in a study of Norway and Denmark, show that Scandinavian sport policy—characterised by strong public support, municipal responsibility, and significant influence from the voluntary sport sector—has resulted in facilities largely designed according to the requirements of sport organisations. These facilities have typically followed federation regulations and are primarily oriented towards competitive sport and club members. This standardisation arguably produces a normative sport landscape (37), in which the quantification and regulation of facilities require the spatial parameters of the physical environment to be as uniform as possible. As Vertinsky and Bale (37) further note, the enclosure of sporting spaces may reinforce hierarchical boundaries regarding who is recognised as an athlete.

At the same time, patterns of participation have changed (8, 38). Whereas organised sport dominated in the 1980s, many individuals today engage in unorganised physical activity. Since the 1990s, the field has become increasingly complex, with new associations and commercial actors promoting alternative cultures and lifestyles. This shift is linked to rising participation among older people and women, many of whom do not use traditional facilities such as football pitches or athletics tracks as frequently [see also (39)]. Instead, there is growing demand for flexible, user-oriented spaces, often provided by non-public actors. Despite this, public investment continues to favour traditional facilities. Regional differences are also evident: multi-functional centres and private studios are concentrated in larger cities, offering more adaptable spaces than standardised, competition-oriented public facilities (8). Another perspective, highlighted by Eime et al. (25), sport facilities not only offer opportunities for sport activities but also play a role in being the social anchor within communities and increase social capital [see also (40)].

From this perspective, the production of sport facilities can be understood as a set of power-laden processes, where competing interests are reflected in the planning, design, construction and use of sporting spaces (37).

Although the sport movement in Sweden has begun adopting new ways of thinking, the facility issue remains problematic, especially in urban regions. Generally, when considering all (organised) sports as a collective, the sport facility area per capita has decreased over the past decades [e.g., (9)]. Simultaneously, studies show that the perceived lack of facilities is higher than the actual lack, which could mean that ‘complaining about the lack of space takes up more time than trying to find solutions’. In addition, the number of bookings is higher than the actual use. Similar tendencies have been shown in other countries, for instance, when investigating Danish sport facilities (41). There is currently limited knowledge about how sport halls are actually used in practice in Sweden. While many sport clubs and federations report insufficient access to facility time, this raises the question of whether more efficient use of existing facilities could help address the perceived shortage. Building on the Danish experiences, the Swedish Research Council for Sport Science (CIF) initiated a pilot project in collaboration with Linköping Municipality and Linköping University. The project aims to examine how similar methods can be applied in a Swedish context and to increase knowledge about facility usage nationwide, using cameras and AI to register activity in sport halls (42). The findings from the project point to discrepancies between booked and actual use. While sport halls were heavily used during weekday evenings and weekend mornings, large amounts of booked time during daytime, late evenings, school holidays, and vacation periods remained unused, highlighting the importance of distinguishing between bookings and actual utilisation when assessing facility shortages (43).

The utilisation pespective is relevant in connection to the facility provision. As mentioned above, there is a long tradition of significant public sector support and subsidising the construction and operations to sport facilities (8, 44), which is expected to create public value in terms of social, health, and democratic advantages for society (44). Based on this, Iversen, Høyer-Kruse and Forsberg (45) state that: “it matters to which extent which bookings, usage, and attendance are valid indicators of the level of public value produced” (p. 593). They discuss these different ways of measuirng the utilisation of sport facilities, and concludes that providing detailed information on usage, rather than bookings (and to some degree attendance), seem to be important for creating learning, steering, control, accountability and legitimacy.

To sum up, although not exhaustive, this section highlights the complexity of evaluating and understanding sport facility provision. There are multiple perspectives and “truths” regarding distribution, proximity, accessibility, utilisation, design requirements, priorities, and related aspects. The research overview presented above aimed to highlight the complexity that the actors involved are required to navigate.

Zhao et al. ((32), p. 160) highlight that “the urban built environment encompasses diverse spaces that facilitate physical activity and sport participation, ranging from purpose-built sport facilities to public recreational spaces and active transportation infrastructure”. They categorise these environments into three key types based on their primary function and level of organisation: sport-specific facilities, public recreational spaces, and active transportation and urban infrastructure. Sport-specific facilities, such as gyms, swimming pools, stadiums, and basketball courts, provide designated spaces for structured or organised forms of sport participation, supporting formal training, competitive events, and recreational engagement.

In this article, the primary focus is on sport-specific facilities, mainly for organised sport, although the research background adopts a broader perspective on sport and physical activity environments.

## 3 Methodology and analytical framework

### 3.1 Research aim, context, and design

As stated earlier, the issue of sport facilities is characterised by a range of challenges, uncertainties, and conflicting viewpoints. Is there a shortage of facilities, and if so, in what ways and for what reasons? What different ways of measuring, understanding, and experiencing this issue exist? What needs and conditions can be identified, and what developments may emerge in light of current trends? Whose interests are prioritised, and who is responsible for facility provision and development? These questions have guided the aim of this article, which is to explore how sport facility provision in urban Sweden is shaped by different perspectives, competing institutional logics, and fragmented governance arrangements, as articulated by key actors across the sport movement, municipal-sector organisations, and knowledge-producing bodies. The study does not seek to determine objectively whether Sweden faces a facility shortage, whether increased provision leads to higher participation, or whether existing facilities correspond to contemporary activity patterns. Rather, it explores how such questions are understood, framed, and negotiated by actors occupying different positions within the sport facility policy field.

The complexity of this study lies in its ambition to identify and connect diverse perspectives, facts, perceptions, and interests related to the urban sport facility landscape. To achieve this, the study is designed as an exploratory qualitative project based on document analysis and five semi-structured interviews with key informants representing central stakeholders.The document material consists of surveys and policy-related documents produced by national umbrella organisations and public authorities responsible for various aspects of sport facility provision or knowledge. As organisational products, they were produced for specific strategic, policy, or advocacy purposes and therefore represent institutional perspectives. The document analysis was consequently used not only as a source of factual information but also as a source of insight into how different actors frame sport facility issues.

As highlighted throughout this article, sport policy in Sweden has long been characterised by close ties between national and local governments on the one hand and voluntary, non-profit, membership-based club sport on the other. Fahlén and Stenling (46) describe the policy coalition that directs, influences, and is influenced by policies concerning sport facility provision in Sweden, namely the national government, organised sport at the national level (RF), municipalities, and organised sport at the local level (sport clubs).

The material presented in this article constitutes the first phase of a two-year project. The second phase builds on these findings and aims to analyse sport clubs' experiences and perceptions of the current facility situation in urban contexts, as well as their willingness and ability to adapt. This latter phase thus focuses on the grassroots level, whereas the present article draws on material from the comprehensive national level and therefore does not encompass the entire policy coalition described by Fahlén and Stenling (46).

The interviews and policy-related documents primarily focus on four national umbrella organisations and public authorities: the Swedish Sports Confederation (RF), the Swedish Association of Local Authorities and Regions (SKR), the Swedish Research Council for Sport Science (CIF), and the Swedish Association of Local Authority Leisure and Cultural Services (SFK). These actors hold different roles in relation to sport facilities.

In addition, two further interviews were conducted: one with a representative from the Ministry of Social Affairs (Swedish Government) responsible for sport-related issues, and one with a representative from the National Board of Housing, Building and Planning (Boverket) responsible for the government assignment on sport facilities. These interviews had a different focus and purpose and are therefore not included in the results section.

As these organisations play a crucial role in the analysis, a brief description of each is provided in Appendix A.

In the results section, five key informants from four of the above-mentioned organisations are referenced:

- RF1: Head of Department, Plats för Idrott (Space for Sport), the Swedish Sports Confederation (RF).
- SKR1: Senior Advisor, Sport and Leisure, the Swedish Association of Local Authorities and Regions (SKR).
- CIF1: Project Coordinator, Sport Facilities, the Swedish Research Council for Sport Science (CIF).
- CIF2: Operations Coordinator for Sport and Outdoor Facilities, the Swedish Research Council for Sport Science (CIF).
- SFK1: Board Member (and Director of Culture and Leisure), the Swedish Association of Local Authority Leisure and Cultural Services (SFK).

It is important to recognise that the organisations represented in this study occupy different positions within the sport facility policy field and therefore bring different institutional perspectives to the issue. RF primarily acts as an advocacy organisation representing the interests of organised sport and the needs of its member federations and clubs, while SKR represents municipalities and local authorities, which are largely responsible for planning, financing, operating, and maintaining sport facilities. SFK functions as a professional network for municipal practitioners, whereas CIF has a governmental mandate to support and disseminate research-based knowledge and policy evaluation. Consequently, the perspectives expressed by the interviewees should not be understood as neutral descriptions of sport facility conditions, but rather as interpretations shaped by the institutional roles, responsibilities, and interests of the organisations they represent.

The purpose of the interviews was not to capture the full range of perspectives within Swedish sport facility provision. Rather, the interviewees were selected as elite informants occupying strategic positions within organisations that play central roles in national policy development, knowledge production, advocacy, and municipal coordination. The study therefore provides insight into how influential actors at the national level understand and frame contemporary sport facility issues. While the number of interviews is limited, the selection was purposive and aimed at capturing key institutional perspectives rather than achieving representativeness. Moreover, because most organisations represented are located in Stockholm and operate primarily at the national level, the findings may under-represent regional and local variations in how sport facility challenges are experienced and prioritised.

### 3.2 Data collection and analysis

The interviews were conducted in a semi-structured format. To capture the key aspects of the study's aim, the interview guide was organised around a set of overarching questions or themes. The questions were intentionally broad and far-reaching in order to capture a wide range of perspectives and to avoid steering respondents in a particular direction. The sequence of questions varied depending on the flow of each interview. Follow-up questions were used to explore specific themes or responses in greater depth and to capture variation between respondents.

The interviews were conducted in March and April 2026 and lasted between 65 and 88 min. All interviews were carried out via Microsoft Teams and recorded.

The empirical material was analysed using a stepwise qualitative approach combining elements of qualitative content analysis and reflexive thematic analysis. Interview data were systematically coded and organised into analytically derived categories and subcategories. This process was guided by a reflexive thematic analysis with a primarily inductive orientation (47, 48), aiming to identify recurring patterns of meaning across respondents while remaining sensitive to the diversity of perspectives represented by different organisations.

Although the interviews were structured around overarching topics, no predefined theoretical framework or analytical categories were imposed during data collection or in the initial stages of analysis. Instead, the analysis proceeded iteratively, allowing themes to emerge through close and repeated engagement with the empirical material.

All interviews were transcribed verbatim and read multiple times to ensure familiarisation with the data. In the first phase of analysis, segments of text were coded with attention to how respondents described:

- institutional responsibilities,
- provision and shortage of facilities,
- current conditions and perceived challenges,
- priorities and developments in facility planning, and
- future needs and trajectories.

These initial codes were then grouped into broader thematic categories. While these categories were partly aligned with the topics covered in the interview guide, they were further refined and reorganised through constant comparison across interviews, enabling differences and similarities between actors to be systematically explored.

In a subsequent phase, the analysis moved from a more descriptive categorisation toward a higher level of abstraction. Themes were condensed and rearticulated into analytically oriented themes, which form the headings in the result section:

- Sport facilities as a re-emerging and contested policy issue
- The notion of facility shortage: shared perception, divergent meanings
- Availability, suitability, and structural mismatch
- Responsibilities and the complexity of multi-level governance
- Standardisation, specialisation, and accumulated renovation needs
- Knowledge production, measurement, and the limits of quantification
- Future development and emerging challenges

To further contextualise the interview material, relevant publications produced by the organisations represented by the respondents were analysed alongside the interview data. These sources were used to corroborate, nuance, and situate interview statements within broader institutional discourses.

Finally, in the discussion section, the empirically derived themes were interpreted through the lens of path dependency and new institutional theory, enabling a theoretically informed analysis of stability, change, and governance dynamics in sport facility provision. An abductive analytical approach was applied, iterating between empirical insights and theoretical perspectives. More specifically, the analysis began with largely inductive coding of the empirical material, after which theoretical concepts from path dependency and new institutional theory were iteratively used to reinterpret and refine the emerging themes. The new institutional theory could be used to analyse how sport facilities are understood, prioritised, and governed in contemporary urban contexts. New institutional theory emphasises how organisations and policy fields are shaped by socially constructed rules, norms, and belief systems rather than by efficiency considerations alone (49, 50). The theories of path dependency and institutional inertia emphasise how past decisions, investments, and institutional arrangements create self-reinforcing trajectories that constrain future options (51).

### 3.3 Ethical statement

The project is conducted in accordance with the Swedish Research Council's ethical guidelines. Particular emphasis is placed on research integrity, including reliability, transparency throughout the research process, accountability for all stages of the study, and respect for participants. All informants received sufficient information about the study and their rights and provided written informed consent prior to participation. None of the participants requested full anonymity.

Participation in the study was entirely voluntary, and no personal or sensitive data were collected. All data were processed in accordance with established ethical standards and the General Data Protection Regulation (GDPR).

## 4 Results: sport facilities in Sweden from actor and knowledge perspectives

This section presents results from the document analysis and interviews with representatives from national sport organisations, municipalities, and knowledge-producing bodies. The findings reveal a complex and contested landscape in which sport facilities are framed simultaneously as scarce resources, costly public assets, and politically sensitive instruments for achieving broader societal goals. While certain concerns are widely shared, the results also demonstrate significant variation in how key actors understand responsibilities, needs, and future priorities.

### 4.1 Sport facilities as a re-emerging and contested policy issue

Both the policy documents analysed and the interviewed actors describe sport facilities as having become increasingly salient within Swedish sport and urban governance over the past decade. Several respondents emphasised that sport facilities have always been part of the Swedish sport model, but that their position in municipal planning has gradually changed.

Earlier studies point to structural developments such as urban densification, increased competition for land, and an ageing facility stock—much of which originates from the 1960s and 1970s—as important background factors. At the same time, planning priorities have shifted towards issues such as public health, everyday physical activity, green infrastructure, and social interaction rather than organised sport per se (36).

CIF1 noted that this shift has contributed to increased ambiguity surrounding what sport facilities are expected to deliver. While organised sport historically enjoyed a relatively taken-for-granted position in municipal planning, respondents suggested that sport facilities must now compete with other urban functions and justify their relevance in new ways. From the perspective of sport organisations, this development is often experienced as a loss of status and influence. However, as interest in sport facility challenges has increased the last decade, the Swedish Sports Confederation (RF) has both taken on and, just recently, been assigned an increasingly prominent role.

### 4.2 The notion of facility shortage: shared perception, divergent meanings

A central finding across both documents and interviews concerns the widespread perception that Sweden faces a shortage of sport facilities. Surveys conducted by the Swedish Sports Confederation repeatedly show that a substantial share of non-profit sport clubs experience limited access to space and time, as well as extensive renovation needs (4). These figures are frequently mobilised in policy debates and advocacy efforts to argue for increased public investment.

Representatives of organised sport largely confirmed this perception. RF1 described facility shortages as one of the most pressing challenges faced by sport clubs, particularly in larger cities, arguing that a “lack of time and space” directly constrains clubs' capacity to sustain and develop their activities. According to RF1, this assessment is reinforced through ongoing dialogue with clubs and regional sport organisations. As an illustrative example, RF1 referred to the Stockholm sport district: when local sport federations were asked to identify the most important issues they wanted RF to prioritise in the coming year, the response was unequivocal—facility provision ranked first, second, and third. At the same time, RF1 emphasised a personal reluctance to use the term “facility shortage,” noting that it can be overly simplistic. Instead, RF1 argued that it is more analytically useful to discuss demand, expectations, supply, and how well these dimensions align. However, this is rarely reflected in the rhetoric of the Swedish Sports Confederation (RF).

Also, interviewees from research-oriented and knowledge-producing organisations offered more nuanced assessments. CIF2 emphasised that the question of shortage is difficult to answer in general terms, noting that there is no agreed definition of what constitutes a shortage, for whom, or in relation to which types of facilities. CIF1 similarly argued that shortages are unevenly distributed across sports, age groups, and local contexts, and that some needs are far more visible in public debate than others.

Municipal perspectives were characterised by caution. SKR1 acknowledged that there are unmet needs related to sport facilities, but warned that the term “shortage” risks oversimplifying a highly complex issue. From this viewpoint, framing the situation as a general shortage may obscure more fundamental questions about what types of facilities are required, what purposes they should serve, and how scarce public resources ought to be prioritised.

### 4.3 Availability, suitability, and structural mismatch

As mentioned above, several studies included in the document analysis complicate the assumption that Sweden suffers from an overarching lack of sport facilities (4). A report by the Swedish Association of Local Authorities and Regions shows that municipalities have more sport facilities than previously reported and that public expenditure on both construction and operation has increased over time (52). The report also indicates that the relationship between facility provision and levels of sport participation is not straightforward (see also section 2.1).

A government-commissioned mapping of school sport halls provides further nuance. The starting point of the study is that organised sport experiences a shortage of facilities, while at the same time a large number of school sport halls remain unused and are not available for booking during afternoons, evenings, and weekends. While making non-bookable halls available to sport clubs would increase nominal capacity, the study concludes that actual utilisation would likely remain limited, as many of these facilities are small, poorly equipped, difficult to access, or otherwise ill-suited to organised sport (53).

Several studies commissioned by the Swedish Sports Confederation (RF) have examined access to sport facilities across different socio-economic and urban contexts. A study on socio-economy and access shows that residents in socioeconomically disadvantaged areas often have greater access to sport facilities than those in more affluent areas, despite lower participation rates (54). Another study analyses access in newly built neighbourhoods, focusing on ball fields, gymnasiums, and sport halls**,** and compares them with older areas (55). The findings indicate poorer access in new developments, while areas built in the 1960s–70s show the best provision, reflecting shifting planning ideals and increasing land pressure.

Hence, analyses of spatial and socioeconomic patterns, highlighted in both the reviewed documents and the perspectives expressed by interviewees, add another layer of complexity. They suggest that proximity alone does not translate into participation, and that availability does not necessarily translate into usability.

Recent evidence from Sweden further suggests that availability and utilisation should not be conflated. A study based on automated activity measurements in municipal sport halls identified a persistent gap between booked and actual use, indicating that perceived shortages may partly reflect inefficiencies in allocation and utilisation rather than an absolute lack of capacity ((43), see also section 2.2).

Interviewees frequently referred to this situation as a mismatch. From this perspective, perceived shortages are not necessarily equivalent to a lack of physical infrastructure. Rather, they may arise when available facilities do not correspond to users' expectations, schedules, organisational requirements, or activity preferences.

CIF1 argued that there may be “more facilities than we assume”, but that ownership structures, booking systems, sport-specific requirements, and increased training frequency among teams hinder access for certain sports and groups. While scheduling and booking systems could be made more strategic, “we cannot require a children's team to train at 10 p.m.” Instead, CIF1 pointed to more efficient use through flexible facility design, such as not requiring full-sized pitches for children's teams. Similarly, SKR1 highlighted the need to assess how effectively facilities are used in relation to their full capacity and potential.

SFK1 similarly noted that facility provision often fails to keep pace with evolving practices, resulting in situations where supply and demand develop out of sync. RF1 pointed out that when facilities are built for, or support is provided to, organised sport, solutions often follow familiar and established models. Creating change requires both energy and courage, which makes it easier to opt for conventional solutions such as building a standard sport hall. According to RF1, this tendency also contributes to an imbalance between facility provision and actual needs.

### 4.4 Responsibilities and the complexity of multi-level governance

Responsibility for sport facilities emerged as a recurring and contested theme in the interviews. Municipalities were widely recognised as the primary actors responsible for planning, financing, building, and operating sport facilities, which has also been recognised in other studies [e.g., (5, 56)].

RF1 stated that municipalities hold “80–90 percent of the power” in facility matters, particularly through spatial planning decisions and booking systems.

At the same time, respondents stressed that responsibility is fragmented across multiple administrative levels and organisational domains. CIF1, as well as SFK1, highlighted that internally divided municipal structures—such as the separation between urban planning and leisure services—often complicate coordination and delay early consideration of sport facilities in planning processes. This fragmentation resonates with findings from a regional study by Region Skåne, which notes the absence of a national sport facility policy and characterises facility provision as a balancing act shaped by multiple, competing needs and interests. Although municipalities play a central role, sport and public health are governed through multi-level governance arrangements**,** involving actors across local, regional, and national levels (57).

The national organisations represented in this study play different roles in this landscape. RF has during the last five to eight years positioned itself as an advocacy actor as well as a provider of expertise and knowledge related to facility needs, primarily through the newly established unit and platform *Plats för idrott* (Space for Sport). SKR primarily represents municipal interests and facilitates knowledge exchange among local authorities. CIF has focused on producing and disseminating research-based knowledge, while SFK supports municipal practitioners through professional networks.

As a consequence of the increasingly widespread perception of a problematic sport facility situation within organised sport—and possibly also because 2026 is an election year in Sweden—the Government (through the Ministry of Social Affairs) decided to prioritise the development of places and facilities during 2026 and 2027 through two targeted initiatives. First, the National Board of Housing, Building and Planning (Boverket) was tasked with supporting municipalities and sport clubs through knowledge provision and process-oriented support. Second, the Swedish Sports Confederation (RF) was allocated SEK 240 million to distribute to sport clubs as co-financing for local construction and renovation projects involving sport halls and other sport facilities.

These policy developments underscore, on the one hand, a shift towards increased national responsibility for sport facility issues and, on the other hand, the fluidity of the governance arrangement. The transfer of responsibility for the national knowledge platform on sport facilities from CIF to Boverket indicates changing institutional mandates and priorities. Several respondents noted that this transition may influence how knowledge is framed and how facility-related issues are positioned within broader planning agendas.

CIF1 emphasised that, compared to CIF's broader assignment—which encompassed multiple target groups and a wide range of facilities for sport, physical activity, and outdoor recreation—the new mandate places a stronger emphasis on traditional sport facilities for organised sport. While increasing physical activity remains an explicit objective, CIF1 argued that the shift reflects a reprioritisation and introduces a preparedness perspective in relation to sport facilities.

### 4.5 Standardisation, specialisation, and accumulated renovation needs

Respondents from several organisations described an ongoing trend towards increasing standardisation and specialisation of sport facilities. CIF1 and SKR1 noted that facilities are increasingly designed for specific sports, disciplines, and performance levels, often in response to federation regulations and evolving competitive norms.

This development was described as ambivalent. While specialisation may enhance quality for certain users, it also reduces flexibility and increases costs. CIF2 argued that almost every sport discipline now requires a distinct type of facility, contributing to perceptions of shortage and limiting opportunities for shared use. Smaller clubs and emerging activities were described as particularly vulnerable in this context. Connected to this, SKR1 noted an increased mental and geographical distance for some groups. SKR1 further suggested that current facility development funding mechanisms tend to advantage larger clubs, as smaller organisations often lack the capacity to access these resources.

Closely related to this issue is the extensive renovation backlog affecting many existing facilities. SFK1 referred to this as an accumulated maintenance debt, resulting from decades of incremental repairs rather than comprehensive renewal. RF1 emphasised that preserving and upgrading existing facilities may be more critical than constructing new ones, especially given rising operational costs and constrained municipal budgets. Several respondents suggested that the growing need to renovate and maintain ageing facilities increasingly constrains the resources available for experimenting with alternative facility concepts or new forms of provision.

### 4.6 Knowledge production, measurement, and the limits of quantification

The demand for quantitative data and key indicators featured prominently in the interviews. Several respondents reported that municipalities increasingly seek benchmarks and numerical guidance—such as how many sport halls are “needed”—to support planning decisions and justify investments. RF1 noted that such requests are common in dialogues with and between municipalities and sport organisations.

At the same time, interviewees expressed reservations regarding the role of quantification. CIF2 pointed out that much of the data currently available reflects perceptions rather than actual use, and that robust, comparable datasets are lacking. SFK1 questioned how utilisation figures should be interpreted, arguing that they often say little about quality, inclusion, or user experience.

SKR1 emphasised that while indicators and visualisation tools can support decision-making, they must be complemented by contextual knowledge and qualitative judgement. Several respondents stressed that numbers alone cannot capture the complex social and organisational dimensions of sport facility provision.

It is worth noting that an increasing number of tools have recently been developed to support primarily municipalities, but also sport organisations, in sport facility planning and decision-making. For instance, the Swedish Sports Confederation (RF) has developed a method for conducting geographical analyses of sport facilities at the municipal level. As stated by RF, through the use of mapping, a more comprehensive overview can be generated, providing a basis for discussing differing conditions across sports and between urban areas. The Swedish Association of Local Authorities and Regions (SKR), as part of the Council for the Promotion of Municipal Analysis *(*Rådet för främjande av kommunala analyser*)*, provides the previously mentioned Kolada database, as well as a new visualisation tool linked to this.

### 4.7 Future development and emerging challenges

Looking ahead, respondents anticipated continued tension surrounding the future development of sport facilities. Environmental sustainability, climate adaptation, and rising operational costs were frequently mentioned as key challenges. SKR1 and CIF1 questioned whether energy-intensive facilities—such as indoor ice rinks—will remain viable in the long term.

Others pointed to potential innovation, including adaptive reuse of existing buildings, technological solutions, and new forms of collaboration between sectors. For instance, SFK1 referred to “smartness” as a guiding principle, encompassing smart operational approaches, sustainability, multifunctionality, the reuse of facilities, efficiency, and the use of sensors. To a large extent, this entails adopting a holistic perspective on sport facility provision.

At the same time, several respondents cautioned that both municipal organisations and the sport movement tend to rely on established models and routines, making change slow and contested. RF1 believes that municipalities will continue to prioritise traditional sport spaces, such as sport halls and ball fields, not least because they can be used by multiple target groups. CIF2, however, identified positive trends: “We can already see signs of change, and I hope we will witness a development that dares to accommodate a wider range of perspectives.”

Overall, the results depict a policy field characterised by competing perspectives, shifting responsibilities, and unresolved questions about what sport facilities are expected to deliver in contemporary urban contexts.

## 5 Discussion

This study set out to explore how sport facility provision in urban Sweden is shaped by multiple perspectives, institutional arrangements, and governance dynamics. The findings from the interviewed national actors and the analysed documents portray a policy field characterised not by a single, clearly defined problem, but by conceptual ambiguity, institutional fragmentation, and competing interpretations of need. By engaging with path dependency and new institutional theory, and by situating the findings within existing research, several key insights emerge.

A first important observation is that sport facility provision is shaped by institutionalised ways of thinking about sport, space, and public value. New institutional theory emphasises how organisations and policy fields are structured by taken-for-granted norms, rules, and belief systems rather than purely rational or efficiency-driven considerations (49, 50). In this study, such institutionalised understandings are visible in the persistence of standardised facility models and in the framing of needs primarily in terms of organised sport. As previous research has shown, Scandinavian sport systems have historically prioritised facilities aligned with federation requirements and competitive structures. The findings suggest that these patterns continue to influence how key national actors understand sport facility provision, even as participation becomes more diversified and increasingly includes informal or unorganised forms (8, 25).

At the same time, the results highlight the continuing relevance of path dependency. Past investments, planning ideals, and institutional arrangements shape current possibilities by creating self-reinforcing trajectories (51). The extensive renovation backlog described by respondents illustrates how facilities built under earlier welfare-state conditions continue to structure budgets, spatial configurations, and planning priorities. As municipalities allocate resources to ageing facility stocks, existing investments generate increasing returns, making alternative forms of provision increasingly difficult and costly to pursue. Consequently, historical infrastructure investments based on traditional understandings of sport shape not only material conditions but also future policy options. Yet path dependency is not solely material; it is also cognitive. Established ideas of what constitutes a “proper” sport facility reinforce institutional inertia and can hinder the adoption of more flexible, multifunctional, or resource-efficient solutions, even when such approaches may be better suited to contemporary urban conditions (9, 36). Respondents repeatedly referred to the tendency to choose familiar solutions, such as standard sport halls, because these are politically recognised, administratively manageable, and aligned with existing organisational routines. This illustrates how institutional inertia is reproduced through everyday decision-making processes rather than merely through formal regulations.

A second key finding concerns the discrepancy between perceived shortage and empirical complexity. While all respondents acknowledged some form of shortage, the results suggest that this notion is perceived and articulated in different ways by the actors represented in this study. This aligns with previous research showing that the relationship between facility provision and participation is neither linear nor uniform (15, 18). Rather than reflecting a simple lack of facilities, the challenges identified in this study relate to issues of distribution, suitability, accessibility, and use. The recurring reference to “mismatch” illustrates how available facilities do not necessarily correspond to the needs of different sports, user groups, or urban contexts. An important distinction emerging from the findings is that between needs and demand. The sport movement frequently articulates a need for additional facilities, yet several respondents questioned whether perceived shortages always reflect an absolute lack of infrastructure. Instead, they may also be influenced by expectations regarding particular facility types, preferred locations, booking routines, and desirable time slots. Seen in this way, some facility challenges may be understood as mismatches between demand patterns, organisational expectations, and the available supply rather than as straightforward deficiencies in the facility stock.

This interpretation also resonates with findings from neighbouring Nordic countries. For example, data from the Norwegian *Ungdata* survey indicate relatively high levels of satisfaction with local sport facility provision among young people (58). This suggests that comparatively high levels of user satisfaction may coexist with claims of facility shortages, indicating that perceptions of shortage should not necessarily be interpreted as straightforward indicators of inadequate provision but rather as socially and institutionally mediated understandings of need.

Moreover, it is important to connect this discussion to studies showing that proximity and availability alone do not guarantee increased participation and that social, cultural, and organisational factors are equally important (24, 25). It also underlines the limitations of treating sport facilities as a purely technical or quantitative issue. As several respondents emphasised, numerical indicators and benchmarks risk oversimplifying complex processes and may obscure qualitative aspects such as inclusivity, usability, and social value. Recent Swedish utilisation research points in a similar direction. While digital activity measurements may improve knowledge about actual facility use, researchers also stress that data require contextual interpretation and should complement rather than replace professional judgement and qualitative assessments (43).

A third insight relates to governance complexity and institutional fragmentation. Sport facility provision in Sweden is characterised by multi-level governance arrangements involving municipalities, national agencies, sport organisations, and knowledge-producing bodies. While municipalities retain primary responsibility, the distribution of roles is fluid and subject to change. The material suggests that fragmentation may contribute to coordination challenges, reflecting the broader institutional context in which sport facilities are embedded. As previous studies have highlighted, Nordic sport systems are characterised by close interdependencies between public authorities and voluntary sport organisations (5, 46). The findings suggest that these relationships remain central but are increasingly contested, particularly in urban contexts marked by competing land-use priorities.

It is also relevant to consider the specific institutional context in which these actors operate. Sweden is a relatively small country, and many of the key actors responsible for sport facility issues are concentrated in Stockholm. On the one hand, this may contribute to a metropolitan bias, where perspectives rooted in larger urban areas risk dominating the discourse. On the other hand, it facilitates regular interaction and close communication among actors, who are familiar with each other and frequently meet in formal and informal settings, such as reference groups and collaborative initiatives. As a result, there appears to be a relatively high degree of shared understanding regarding the overall challenges associated with sport facility provision. At the same time, actors position themselves differently in relation to these challenges, reflecting the interests and institutional roles they represent

At the same time, the study identifies tendencies towards institutional reproduction. Despite awareness of inefficiencies and changing needs, actors often fall back on established solutions, such as traditional sport halls and standardised facilities. As discussed above, this tendency can be understood through the lens of path dependency, whereby previous investments and institutional arrangements shape present choices. Yet the findings may also be interpreted through the lens of institutional isomorphism (50), as organisations within the sport facility field appear to adopt similar practices in response to coercive pressures from regulations and funding arrangements, normative pressures associated with professional expertise and planning practice, and mimetic pressures that encourage the replication of familiar and legitimate facility models under conditions of uncertainty. Municipalities, sport organisations, and professional networks operate within a shared policy field in which certain facility models are regarded as legitimate and appropriate. Consequently, new investments often resemble existing ones, not necessarily because they represent the most effective solution, but because they align with established expectations, professional norms, and sport-specific standards. Such processes reinforce stability but may also hinder innovation and adaptation.

Finally, the study returns to the question of sport facility shortage, which serves as a central entry point for both policy debate and this article. The findings show that all respondents recognise some form of shortage, but importantly, not in the simplified sense often articulated by the sport movement. Instead, shortage emerges as a multidimensional and context-dependent phenomenon, shaped by structural mismatches, changing participation patterns, institutional constraints, and competing priorities.

This has important implications. The dominant narrative—frequently promoted by the Swedish Sports Confederation—emphasises the need for increased construction. While additional investment may in some cases be justified, such an approach risks overlooking alternative strategies. As highlighted in both the results and previous research, more critical questions concern how existing facilities are used, how needs are defined, and how sport practices themselves evolve. For instance, increased training frequency, early specialisation, and rising expectations within organised sport contribute to escalating demand for facility time. Addressing these dynamics may require not only new infrastructure but also a reconsideration of organisational practices and norms.

Moreover, greater attention could be given to more flexible and diversified uses of space, including the integration of public and informal environments into sport activities. This aligns with research emphasising the importance of multifunctional and accessible environments for contemporary participation patterns (32, 33).

In conclusion, within the material analysed, shortage emerges not as a singular phenomenon but as a multidimensional and context-dependent concept. The interviewed actors associated it with structural mismatches, changing participation patterns, institutional constraints, and competing priorities. Moving forward, addressing this issue requires not only investments in infrastructure, but also a more nuanced and reflexive approach to how sport facility needs are conceptualised, governed, and negotiated.

Interestingly, the analysed documents–and to some degree the interviews as well–suggest increasing convergence in how sport facility challenges are framed. Although the organisations occupy different institutional positions, their publications increasingly emphasise planning support, evidence-based decision-making, indicators, accessibility, and strategic management. From an institutional perspective, this may indicate a degree of isomorphism within the policy field, as actors employ increasingly similar concepts and problem definitions despite representing different interests.

Some aspects of the Swedish case are likely shaped by the institutional characteristics of the Nordic welfare model. The historically strong role of municipalities in financing, operating, and subsidising sport facilities has created institutional arrangements that differ from those found in more market-oriented systems. However, the findings suggest that processes commonly associated with path dependency, institutional inertia, and standardisation are not unique to the Swedish or Nordic context. Tensions between perceived facility shortages and broader questions of accessibility, suitability, and utilisation have been observed in both Nordic and non-Nordic settings [see, e.g., (15, 25, 45)]. Likewise, research from a range of countries highlights how established facility models tend to persist despite changing participation patterns and growing demand for more flexible forms of provision (8, 26). From this perspective, the Swedish case is perhaps less an exception than an illustration of wider challenges involved in adapting inherited sport infrastructure systems to contemporary patterns of participation and evolving urban conditions.

Nevertheless, this study should be interpreted in light of its methodological scope. The interview material is based on a limited number of elite informants representing organisations with central roles in Swedish sport facility provision at the national level. Consequently, the findings should not be understood as representative of all actors involved in the sector, particularly municipalities, local sport clubs, or other stakeholders operating outside the national policy arena. Rather, the study provides insight into how influential actors frame, interpret, and negotiate contemporary sport facility challenges. Future research should complement these perspectives by examining local-level experiences and governance practices.

## 6 Summarising words

This study set out to explore how sport facility provision in urban Sweden is shaped by multiple perspectives, institutional arrangements, and governance dynamics. The findings demonstrate that while a perceived shortage of sport facilities is widely acknowledged, it is understood in diverse and often conflicting ways. Rather than indicating a simple lack of infrastructure, the results point to structural mismatches, institutional path dependencies, and fragmented governance as key explanatory factors. By integrating empirical insights with institutional theory, the study contributes to a more nuanced understanding of sport facility provision as a complex, negotiated, and context-dependent policy field.

## Data Availability

The datasets presented in this article are not readily available because the datasets generated and analysed during the current study are not publicly available due to the qualitative nature of the material and considerations of participant confidentiality. Requests to access the datasets should be directed to karin.book@mau.se.

## Appendix A

**TABLE A1:**

Organisation	Role	Website/ Reference
Swedish Sports Confederation (RF)	The *Swedish Sports Confederation (RF)* is the umbrella organisation for the Swedish sport movement, representing national federations and local clubs. It promotes participation, supports member organisations, and distributes government funding for sport. In relation to sport facilities, RF plays an advocacy and knowledge-producing role, highlighting needs, inequalities, and development challenges. Its initiative “Plats för idrott” (Space for Sport) works to map access to facilities, develop indicators, and raise awareness of shortages and mismatches. It also provides guidance for planning and aims to strengthen the conditions for inclusive, accessible, and sustainable facility provision across municipalities.	https://www.rf.se/rf-arbetar-med/idrottsanlaggningar-och-idrottsmiljoer
Swedish Association of Local Authorities and Regions (SKR)	The *Swedish Association of Local Authorities and Regions (SKR)* represents Sweden's municipalities and regions, supporting and advocating for local governance and public services. In relation to sport facilities, SKR plays a strategic and advisory role by producing guidelines, policies and knowledge on planning, financing, and maintenance. It also manages the Kolada database, which provides comparative statistics on municipal infrastructure, including sport facilities. Through these functions, SKR contributes to shaping national policy discussions on facility provision, accessibility, and development.	https://skr.se/kulturochfritid/idrottochanlaggningar.7674.html
Swedish Association of Local Authority Leisure and Cultural Services (SFK)	The *Swedish Association of Local Authority Leisure and Cultural Services (SFK)* is a professional network and meeting platform for individuals in leadership positions within the municipal culture and leisure sector. Its main role is to support the exchange of knowledge and experience, facilitate coordination among practitioners working with sport and cultural issues, and strengthen leadership through networks, training programmes, digital seminars, and an annual conference. Sport facilities constitute a key focus area, where members share experiences and develop knowledge related to planning, management, and development.	https://www.svenskalag.se/sfk
Swedish Research Council for Sport Science (CIF)	The *Swedish Research Council for Sport Science (CIF)* is a government-funded body tasked with promoting, supporting, and disseminating research on sport and physical activity in Sweden. It allocates research funding, evaluates sport policy, and produces annual reports that inform public debate and decision-making. In relation to sport facilities, CIF contributes primarily through knowledge production and policy-relevant analysis. It highlights how access to facilities shapes participation patterns, equality, and public health outcomes, and it problematises issues such as distribution, accessibility, and long-term investment needs in sport infrastructure. However, CIF's role in relation to knowledge production around sport facilities is about to change the Swedish government has transferred most of its responsibilities to the *Swedish National Board of Housing, Building and Planning (Boverket)*, see below.	https://centrumforidrottsforskning.se
Swedish National Board of Housing, Building and Planning (Boverket)	The *Swedish National Board of Housing, Building and Planning (Boverket)* is the central government authority responsible for spatial planning, urban development, housing policy, and the built environment in Sweden. It issues regulations, provides guidance, and supports municipalities in planning processes. In relation to sport facilities, Boverket's role has recently been strengthened through a government assignment, in order to strengthen the provision of sport facilities, focused on knowledge production and on supporting municipalities and civil society organisations with expertise and process-oriented guidance in the planning and development of sport facilities.	https://www.regeringen.se/regeringsuppdrag/2026/01/uppdrag-till-boverket-att-stodja-okad-tillgang-till-idrottshallar-och-annan-plats-for-idrott

## References

[1] Iwarsson A. Föreningar tackar nej – på grund av platsbrist. Aftonbladet. (2026). Available online at: https://www.aftonbladet.se/debatt/a/n1L5OQ/foreningar-tackar-nej-pa-grund-av-platsbrist (Accessed May 15, 2026).

[2] Gromer JJ Forssmed. “Största statliga satsningen på decennier”. Idrottsbranschen. (2026). Available online at: https://idrottsbranschen.se/jakob-forssmed-storsta-statliga-satsningen-pa-decennier/ (Accessed May 15, 2026).

[3] Centrum för idrottsforskning [Swedish Research Council for Sport Science]. Idrottsanläggningar – I dag och I Morgon: Om Behov, Tillgänglighet och Konkurrerande Intressen [Sport Facilities – Today and Tomorrow: On Needs, Accessibility, and Competing Interests]. Stockholm: CIF (2022). Rapport 2022:2.

[4] Riksidrottsförbundet [the Swedish Sports Confederation]. På Bana, is och Matta. Anläggningssituationen I Sverige 2026 [on Track, Ice and Mat: The Sport Facility Situation in Sweden 2026]. Stockholm: Riksidrottsförbundet (2026). Available online at: https://www.rf.se/download/18.42dc5ac519ddca8270ec65e3/1777984802347/Pa_bana,_is_och_matta_-_Riksidrottsforbundets_anlaggningsenkat_2026.pdf (Accessed May 7, 2026).

[5] Bergsgard NA Borodulin K Fahlen J Høyer-Kruse J Iversen EB. National structures for building and managing sport facilities: a comparative analysis of the nordic countries. Sport in Society. (2019) 22(4):525–539. 10.1080/17430437.2017.1389023

[6] Norberg JR. Idrottsrörelsen och staten [the sport movement and the state]. In: Lindroth J Norberg JR, editors. Riksidrottsförbundet 1903–2003. Stockholm: Informationsförlaget (2002). p. 181–231.

[7] Zethrin N. Idrottsanläggningar och kommunerna. Perspektiv på beståndens utveckling och effekter. In: Dartsch Norberg Pihlblad, editors. Idrottsanläggningar – I dag och I Morgon: Om Behov, Tillgänglighet och Konkurrerande Intressen. Stockholm: Centrum för idrottsforskning (2022). pp. 53–72. Rapport 2022:2

[8] Rafoss K Troelsen J. Sports facilities for all? The financing, distribution and use of sports facilities in scandinavian countries. Sport in Society. (2010) 13(4):643–656. 10.1080/17430431003616399

[9] Sjöblom P. ‘Idrott åt Alla’ I en Förtätad och Exploaterad Storstad: Breddidrottens Anläggningar och Intressenter I Stockholms Stad 1985–2014. [’Sport for All’ in a Densified and Intensively Developed Metropolitan Area: Grassroots Sport Facilities and Stakeholders in the City of Stockholm, 1985–2014]. Stockholm: Stockholms Idrottsförbund (2015). FoU-rapport 2015:1

[10] Book K. Dags att tänka nytt om platser för idrott och fysisk aktivitet – utbud, tillgänglighet och flexibilitet [time to rethink places for sport and physical activity – range, accessibility and flexibility]. In: Dartsch C Norberg J Pihlblad J, editors. Idrottsanläggningar – I dag och I Morgon: Om Behov, Tillgänglighet och Konkurrerande Intressen [Sport Facilities – Today and Tomorrow: On Needs, Accessibility and Competing Interests]. Centrum för idrottsforskning (2022). pp. 21–51. Rapport 2022:2

[11] Mace E. (2026). *Mer än ord - ge idrotten plats i* Skåne. RF SISU Skåne, 5 May. Available online at: https://www.rfsisu.se/distrikt/skane/nyhetsarkiv/2026/2026-05-05-mer-an-ord—ge-idrotten-plats-i-skane (Accessed May 17, 2026).

[12] Seippel Ø Breuer C Elmose-Østerlund K Feiler S Perényi S Piątkowska M. In troubled water? European sports clubs: their problems, capacities and opportunities. Journal of Global Sport Management. (2023) 8(1):203–225.

[13] Halonen JI Stenholm S Kivimäki M Pentti J Subramanian SV Kawachi I. Is change in availability of sports facilities associated with change in physical activity? A prospective cohort study. Prev Med. (2015) 73:10–14. 10.1016/j.ypmed.2015.01.012 25602907

[14] Chen Y Lin N Wu Y Ding L Pang J Lv T. Spatial equity in the layout of urban public sports facilities in Hangzhou. PLoS One. (2021) 16(9):e0256174. 10.1371/journal.pone.0256174 34473748 PMC8412330

[15] Hoekman R Breedveld K Kraaykamp G. A landscape of sport facilities in The Netherlands. International Journal of Sport Policy and Politics. (2016) 8(2):305–320. 10.1080/19406940.2015.1099556 s. 205.

[16] Wang W Cai Y Xiong X Xu G. Evaluating accessibility and equity of multi-level urban public sports facilities at the residential neighborhood scale. Buildings. (2025) 15(10):1640–1662. 10.3390/buildings15101640

[17] Pouliou T Lowe S Higgs G. Assessing the health impacts of adults’ participation in sports: investigating the role of accessibility to sport facilities. International Journal of Population Data Science. (2019) 4(3). 10.23889/ijpds.v4i3.1216

[18] Karusisi N Thomas F Méline J Chaix B. Spatial accessibility to specific sport facilities and corresponding sport practice: the RECORD study. Int J Behav Nutr Phys Act. (2013) 10:48. 23601332 10.1186/1479-5868-10-48 PMC3641972

[19] Zasimova L. Sports facilities’ location and participation in sports among working adults. European Sport Management Quarterly. (2022) 22(6):812–832. 10.1080/16184742.2020.1828968

[20] Faskunger J Sjöblom P. Anläggningar och andra miljöer för idrott – när, hur och varför är de samhällsnyttiga? [sport facilities and other environments for physical activity – when, how, and why are they socially beneficial?]. In: Idrottens Samhällsnytta. En Vetenskaplig översikt av Idrottsrörelsens Mervärden för Individ och Samhälle (Stockholm: RF) (2017), 191–212*.* FoU-rapport 2017:1

[21] Parks SE Housemann RA Brownson RC. Differential correlates of physical activity in urban and rural adults of various socioeconomic backgrounds in the United States. Journal of Epidemiololgy and Community Health. (2003) 57(1):29–35. 10.1136/jech.57.1.29 PMC1732269 12490645

[22] Hallmann K Wicker P Breuer C Schönherr. Understanding the importance of sport infrastructure for participation in different sports: findings from multi-level modeling. European Sport Management Quarterly (2012) 12(5): 525–544. 10.1080/16184742.2012.687756

[23] Wicker P Breuer C Pawlowski T. Promoting sport for all to age-specific target groups: the impact of sport infrastructure. European Sport Management Quarterly. (2009) 9(2):103–118. 10.1080/16184740802571377

[24] Prins RG Mohnen SM Van Lenthe FJ Brug J Oenema A. Are neighbourhood social capital and availability of sports facilities related to sports participation among Dutch adolescents? Int J Behav Nutr Phys Act. (2012) 9(90):90. 10.1186/1479-5868-9-90 22849512 PMC3479015

[25] Eime RM Harvey J Charity MJ Casey M Westerbeek H Payne WR. The relationship of sport participation to provision of sports facilities and socioeconomic status: a geographical analysis. Aust N Z J Public Health. (2017) 41(3):248–255. 10.1111/1753-6405.12647 28110514

[26] Wicker P Hallmann K Breuer C. Analyzing the impact of sport infrastructure on sport participation using geo-coded data: evidence from multi-level models. Sport Management Review. (2013) 16(1):54–67. 10.1016/j.smr.2012.05.001

[27] Higgs G Langford M Norman P. Accessibility to sport facilities in Wales: a GIS-based analysis of socio- economic variations in provision. Geoforum. (2015) 62:105–120. 10.1016/j.geoforum.2015.04.010

[28] Lotfi S Koohsari MJ. Analyzing accessibility dimension of urban quality of life: where urban designers face duality between subjective and objective Reading of place. Soc Indic Res. (2009) 94:417–435. 10.1007/s11205-009-9438-5

[29] Fathi S Sajadzadeh H Mohammadi Sheshkal F Aram F Pinter G Felde I. The role of urban morphology design on enhancing physical activity and public health. Int J Environ Res Public Health. (2020) 17(7):2359. 10.3390/ijerph17072359 32244358 PMC7178257

[30] McCormack GR Rock M Toohey AM Hignell D. Characteristics of urban parks associated with park use and physical activity: a review of qualitative research. Health Place. (2010) 16(4):712–726. 10.1016/j.healthplace.2010.03.003 20356780

[31] Cohen D Han B Park S Williamson S Derose K. Park use and park-based physical activity in low-income neighborhoods. J Aging Phys Act. (2018) 27(3):334–342. 10.1123/japa.2018-0032 30160585 PMC7494055

[32] Zhao TT Chen SS Mu L Bai Y Collins C Zhang JJ. Staying active in cities: dimensions of urban built environment and mass sport participation. European Sport Management Quarterly. (2026) 26(1):158–183. 10.1080/16184742.2025.2522713

[33] Book K Högdahl E. Equalizer: breaking down the barriers at informal outdoor sport and recreational spaces. Leis Stud. (2022) 43(6):961–977. 10.1080/02614367.2022.2088833

[34] Riksidrottsförbundet [the Swedish Sports Confederation]. Närhet till anläggning gör oss aktivare [Proximity to sport facilities makes us more active]. (2023) Available online at: https://www.rf.se/idrottisamhallet/narhettillanlaggninggorossaktivare (Accessed April 30, 2026).

[35] Giles-Corti B Donovan R. The relative influence of individual, social and physical environment determinants of physical activity. I Social Science & Medicine. (2002) 54(12):1793–1812. 10.1016/S0277-9536(01)00150-2 12113436

[36] Book K. Idrotten I den Fysiska Planeringen [Sport in Spatial Planning]. Stockholm: RF (2016). FoU-rapport 2015:2.

[37] Vertinsky PA Bale J. Sites of Sport: Space, Place and Experience. Florence: Taylor & Francis (2004).

[38] Ibsen B. Idrætsvaner og Faciliteter I Danmark [Sports Participation and Sport Facilities in Denmark]. Copenhagen: Idrættens Analyseinstitut (Idan) [Danish Institute for Sports Studies] (2013).

[39] Limstrand T. Environmental characteristics relevant to young people's Use of sports facilities: a review. Scand J Med Sci Sports. (2008) 18:275–287. 10.1111/j.1600-0838.2007.00742.x 18384494

[40] Seifried C Clopton AW. An alternative view of public subsidy and sport facilities through social anchor theory. City Culture Soc. (2013) 4(1):49–55. 10.1016/j.ccs.2013.01.001

[41] Høyer-Kruse J Iversen EB Forsberg P. Idrætsanlægs Benyttelse og Brugernes Tilfredshed.’ Movements, 7. Odense: Center for Forskning i Idræt, Sundhed og Civilsamfund, Institut for Idræt og Biomekanik, Syddansk Universitet (2017).

[42] Centrum för idrottsforskning [Swedish Research Council for Sport Science]. *Kan idrottshallar nyttjas mer effektivt?* (2024) Available online at: https://centrumforidrottsforskning.se/nyheter/kan-idrottshallar-nyttjas-mer-effektivt-nytt-forskningsprojekt-i-linkoping/ (Accessed April 30, 2026).

[43] Iacobaeus H Kazlou A Toll D Ekholm D. Kommunala Strategier för Effektiv Användning av Lokaler: Aktivitetsmätningar I Idrottshallar [Municipal Strategies for the Efficient Use of Facilities: Activity Monitoring in Sport Halls]. Norrköping: Centrum för kommunstrategiska studier (2026) 2026:2. Rapport 2026:2. 10.3384/9789181186451

[44] Ibsen B Nichols G Piątkowska M Nagel S Llopis- Goig R Elmose-Østerlund K. What can explain the differences between European countries’ public policies for sports clubs? International Journal of Sport Policy and Politics. (2022) 14(3):1–17. 10.1080/19406940.2022.2052148

[45] Iversen EB Høyer-Kruse J Forsberg P. How do we measure? Perspectives for learning, steering, and accountability when measuring the utilisation of sports facilities. Managing Sport and Leisure. (2024) 31(3):591–604. 10.1080/23750472.2024.2356187

[46] Fahlén J Stenling C. Sport policy in Sweden. International Journal of Sport Policy and Politics. (2016) 8(3):515–531. 10.1080/19406940.2015.1063530

[47] Braun V Clarke V. Using thematic analysis in psychology. Qual Res Psychol. (2006) 3(2):77–101.

[48] Braun V Clarke V. Reflecting on reflexive thematic analysis. Qual Res Sport Exerc Health. (2019) 11(4):589–597. 10.1080/2159676X.2019.1628806

[49] Meyer JW Rowan B. Institutionalized organizations: formal structure as myth and ceremony. Am J Sociol. (1977) 83(2):340–363.

[50] DiMaggio PJ Powell WW. The iron cage revisited: institutional isomorphism and collective rationality in organizational fields. Am Sociol Rev. (1983) 48(2):147–160. 10.2307/2095101

[51] Pierson P. Increasing returns, path dependence, and the study of politics. Am Polit Sci Rev. (2000) 94:251–267. 10.2307/2586011

[52] Sveriges Kommuner och Regioner [the Swedish Association of Local Authorities and Regions]. Idrottsanläggningar nu och för Framtiden. Nationell Statistisk, Verktyg för Analys, Kunskap för Utveckling [Sports Facilities for the Present and the Future: National Statistics, Analytical Tools, and Knowledge for Development]. Stockholm: Sveriges Kommuner och Regioner (2025). Available online at: https://extra.skr.se/download/18.61c1455019afccb5d351bd11/1765529490267/A4_SKR_Idrottsanlaggningar_webbpdf.pdf (Accessed April 15, 2026).

[53] Centrum för idrottsforskning [Swedish Research Council for Sport Science], Spacescape. Idrottsanläggningar på Skolfastigheter. Analys av Idrottsrörelsens Tillgång Till Anläggningar på Skolor [Sport Facilities on School Premises: An Analysis of the Sport Movement's Access to School-Based Facilities]. Stockholm: Centrum för idrottsforskning 2025:1 (2025). Available online at: https://centrumforidrottsforskning.se/wp-content/uploads/2026/03/Idrottsanlaggningar-pa-skolfastigheter_0-1.pdf (Accessed April 15, 2026).

[54] Riksidrottsförbundet [the Swedish Sports Confederation]. Tillgång Till Idrott och Socioekonomi [Access to Sport and Socioeconomic Conditions]. Stockholm: RF/Spacescape (2025a).

[55] Riksidrottsförbundet [the Swedish Sports Confederation]. Tillgång Till Idrott I Nybyggda Områden [Access to Sport in Newly Developed Areas]. Stockholm: RF/Spacescape. (2025b).

[56] Norberg JR. A mutual dependency – the nordic sport and the state. In: Meinander H Mangan JA, editors. In the Nordic World. Sport in Society. London: Frank Cass (1998). p. 115–134.

[57] Region Skåne [The regional authority of Skåne]. Idrottsanläggningar i Skåne—Förutsättningar och möjligheter för mellankommunal samverkan (förstudie) [Sport facilities in Skåne: Preconditions and opportunities for inter-municipal cooperation (pre-study)]. Malmö: Region Skåne. Available online at: https://utveckling.skane.se/siteassets/publikationer/idrottsanlaggningar-i-skane—mellankommunal-samanvandning.pdf (Accessed April 30, 2026).

[58] Bakken A. Ungdata 2025. Nasjonale Resultater. NOVA Rapport 4/25. Oslo: NOVAOsloMet (2025).

